# 

**Published:** 1891-09

**Authors:** 


					236 American Journal of Dental Science.
Communications.
AMERICAN MEDICAL ASSOCIATION.
Philadelphia, August 14th, 1891.
Dear Doctor:
Kindly insert the accompanying news item in the next
issue of your esteemed journal. The numerous letters I
receive from physicians who seem not to know of the exist-
ence of this method of admission to the Association show that
there is need of more general information on the subject.
Yours very truly,
Richard J. Dunglison, M. D., Treas.
Membership in the American Medical Associa-
tion.?This is obtainable, at any time, by a member of any
State or local Medical Society which is entitled to send
delegates to the Association. All that is necessary is for the
applicant to write to the Treasurer of the Association, Dr.
Richard J. Dunglison, Lock Box 1274, Philadelphia, Pa,,
sending him a certificate or statement that he is in good stand-
ing in his own Society, signed by the President and Secretary
of said Society, with five dollars for annual fees. Attendance
as a delegate at an annual meeting of the Association
is not necessary in order to obtain membership. On receipt
of the above amount the weekly Journal of the Association
will be forwarded regularly.
To Readers and Correspondents.
Communications intended for publication, and exchange journals
and papers, should be addressed to the Editor of The American
Journal of Dental Science, No. 845 N. Eutaw St. Baltimore, Md.
All letters relating to the business of the Journal, or containing cash
remittances, to be directed to Messrs. Snowden & Cowman, No. 9 W.
Fayette Street, Baltimore, Md. Articles lor publication should be re-
ceived by the Editor at least two weeks previous to the first of the
month on which the number in which it is designed for them to appear,
is issued.
The American Journal of Dental Science will contain fifty
pages of reading matter in each number, making a volume
of about Six Hiindred pages,
EXCLUSIVE OF ADVERTISEMENTS

				

